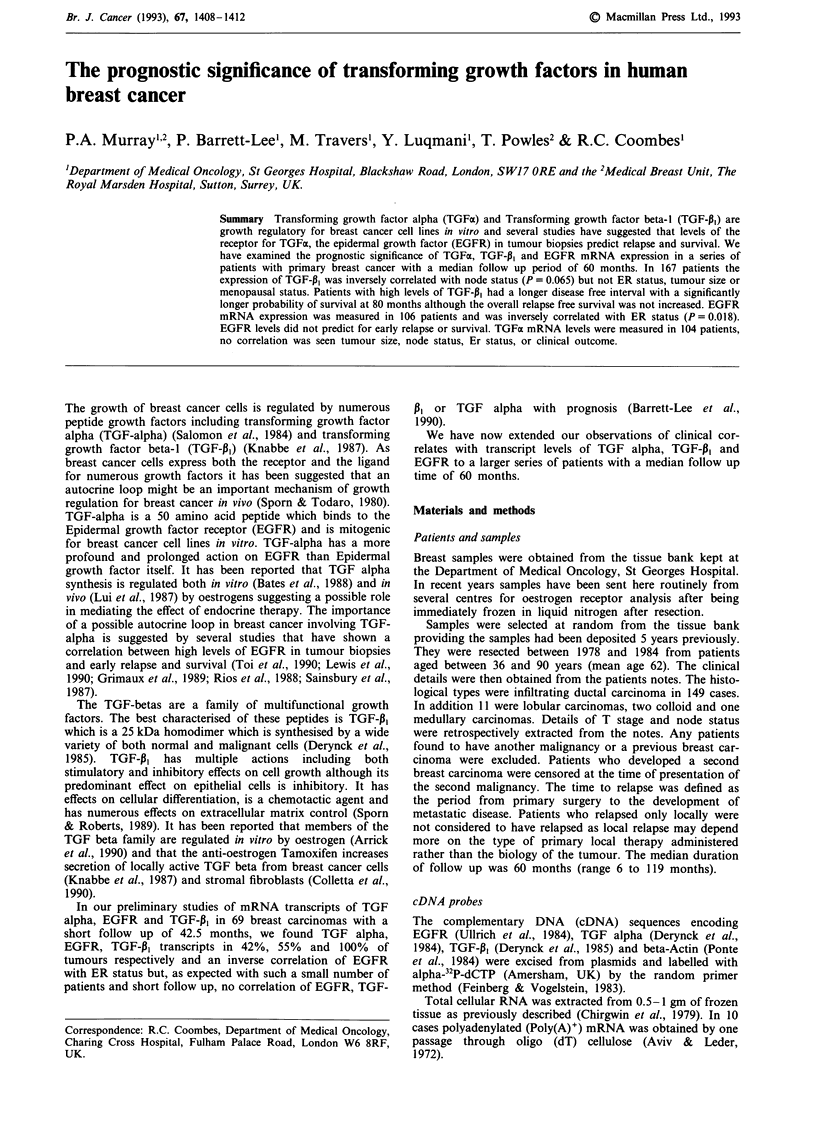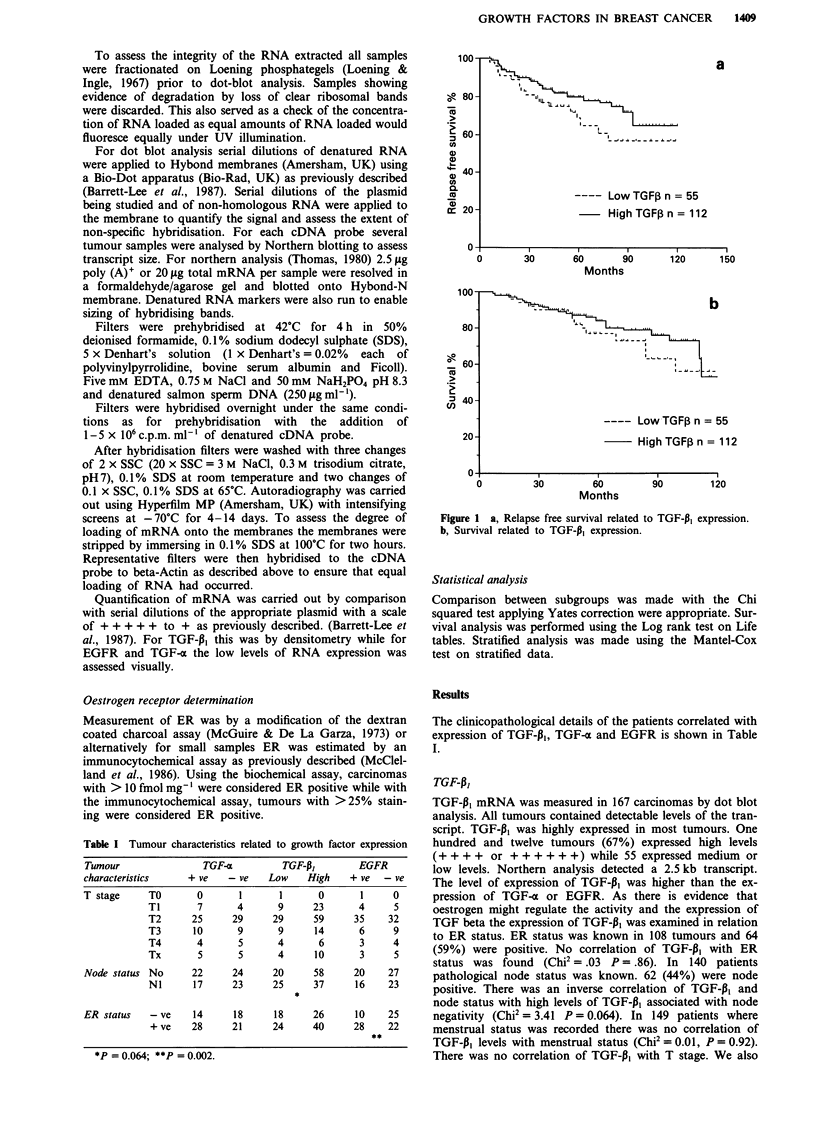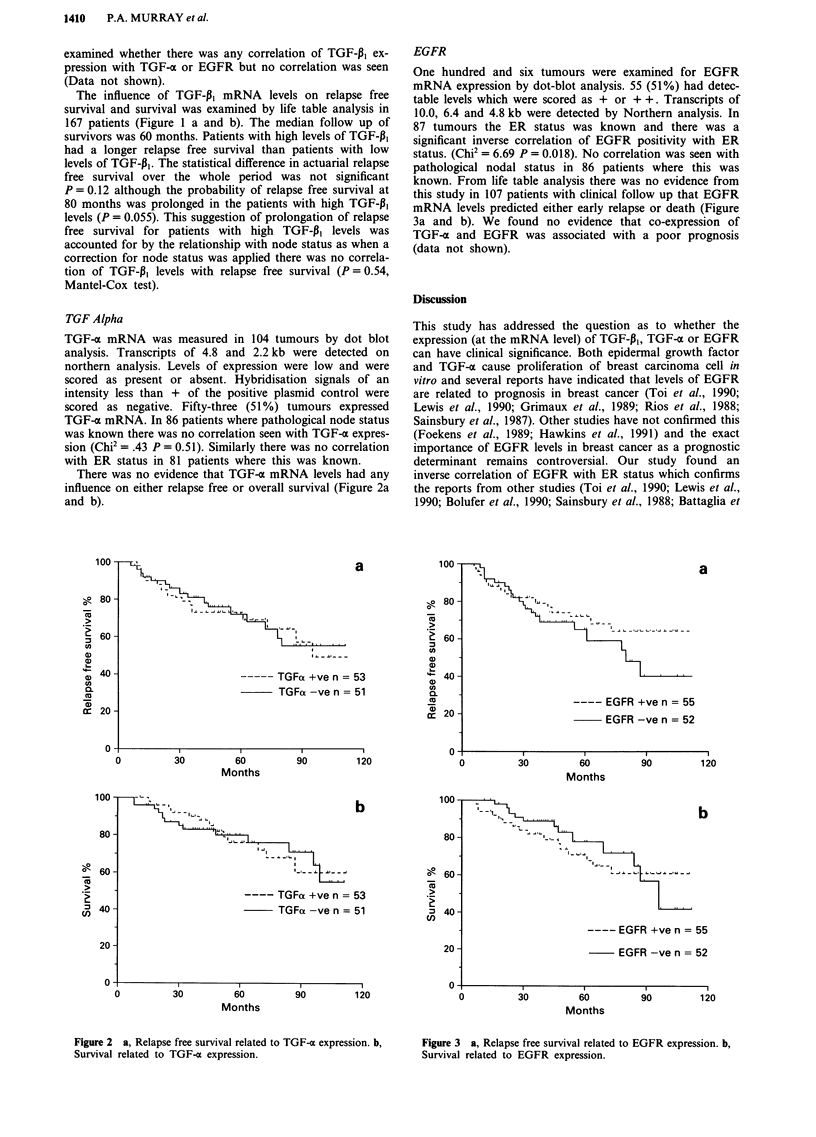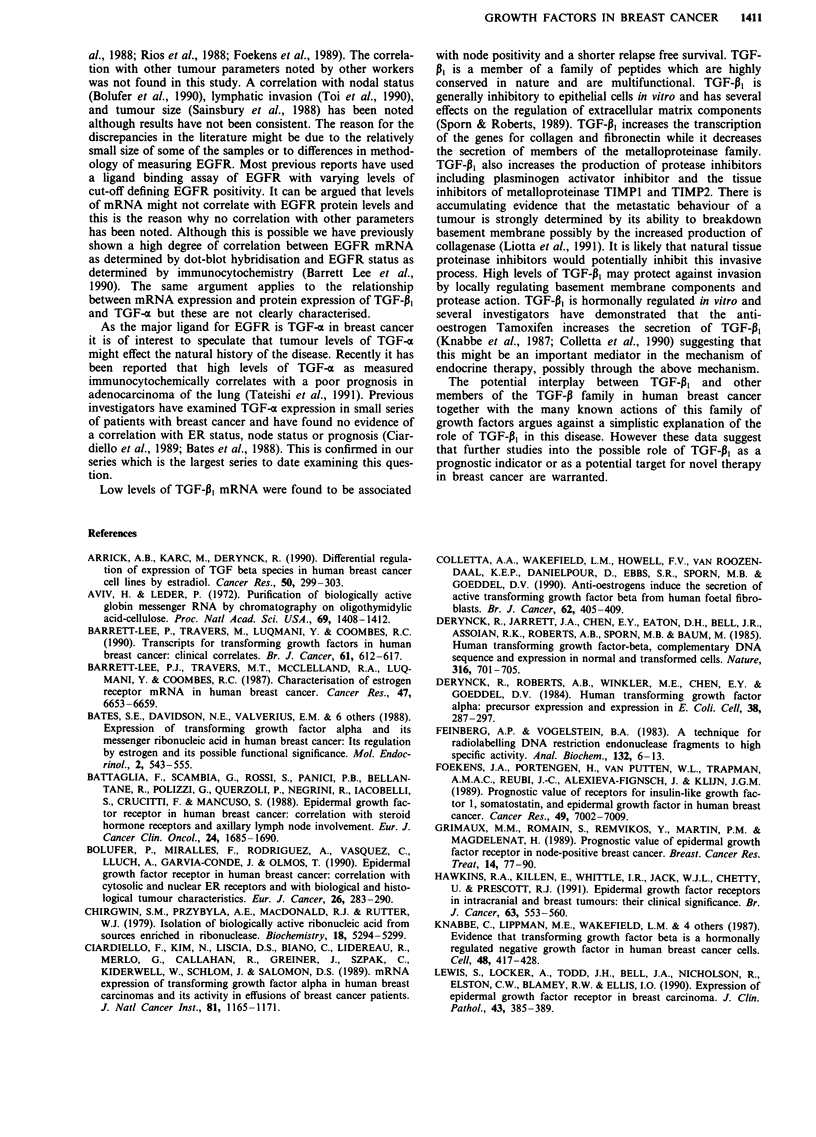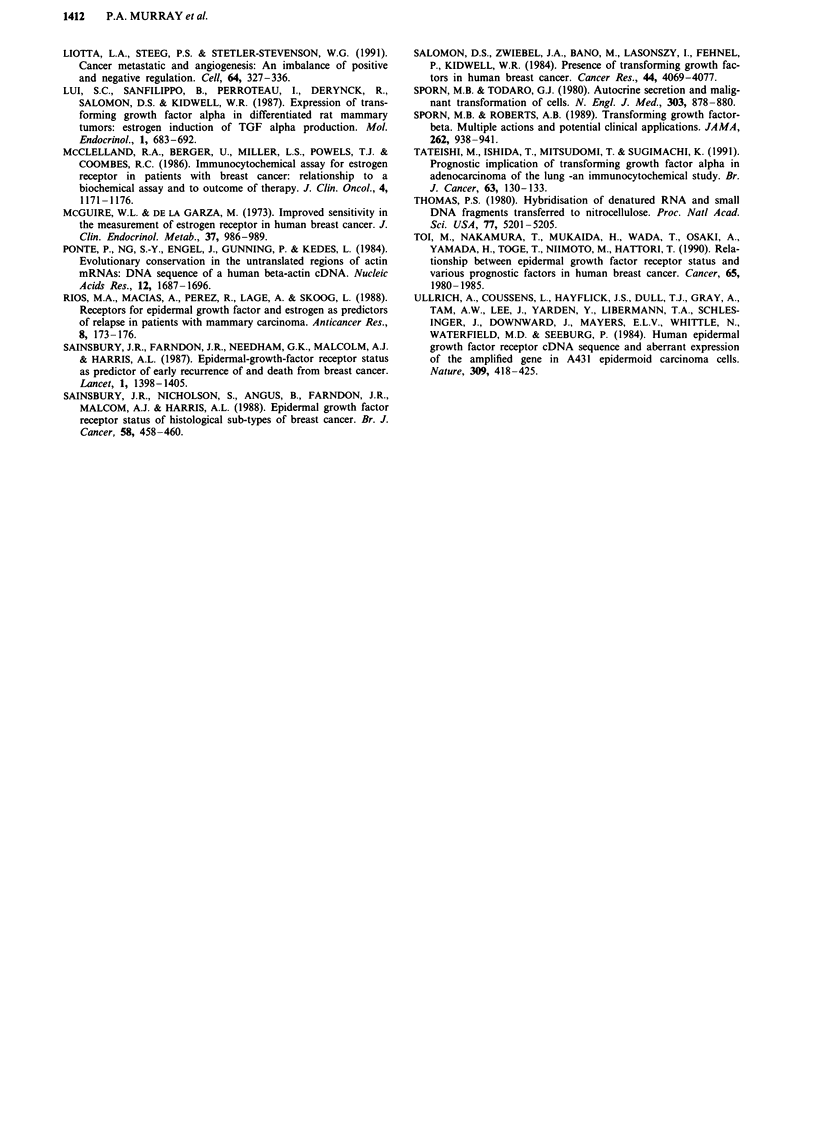# The prognostic significance of transforming growth factors in human breast cancer.

**DOI:** 10.1038/bjc.1993.261

**Published:** 1993-06

**Authors:** P. A. Murray, P. Barrett-Lee, M. Travers, Y. Luqmani, T. Powles, R. C. Coombes

**Affiliations:** Department of Medical Oncology, St Georges Hospital, London, UK.

## Abstract

Transforming growth factor alpha (TGF alpha) and Transforming growth factor beta-1 (TGF-beta 1) are growth regulatory for breast cancer cell lines in vitro and several studies have suggested that levels of the receptor for TGF alpha, the epidermal growth factor (EGFR) in tumour biopsies predict relapse and survival. We have examined the prognostic significance of TGF alpha, TGF-beta 1 and EGFR mRNA expression in a series of patients with primary breast cancer with a median follow up period of 60 months. In 167 patients the expression of TGF-beta 1 was inversely correlated with node status (P = 0.065) but not ER status, tumour size or menopausal status. Patients with high levels of TGF-beta 1 had a longer disease free interval with a significantly longer probability of survival at 80 months although the overall relapse free survival was not increased. EGFR mRNA expression was measured in 106 patients and was inversely correlated with ER status (P = 0.018). EGFR levels did not predict for early relapse or survival. TGF alpha mRNA levels were measured in 104 patients, no correlation was seen tumour size, node status, Er status, or clinical outcome.


Br. J. Cancer (1993), 67, 1408-1412 © Macmillan Press Ltd., 1993

### The prognostic significance of transforming growth factors in human breast cancer

P.A. Murray[1,2], P. Barrett-Lee[1], M. Travers[1], Y. Luqmani[1], T. Powles[2] & R.C. Coombes[1]

[1]*Department of Medical Oncology, St Georges Hospital, Blackshaw Road, London, SW17 0RE and the* [2]*Medical Breast Unit, The Royal Marsden Hospital, Sutton, Surrey, UK.*

**Summary** Transforming growth factor alpha (TGFα) and Transforming growth factor beta-1 (TGF-$\beta_1$) are growth regulatory for breast cancer cell lines *in vitro* and several studies have suggested that levels of the receptor for TGFα, the epidermal growth factor (EGFR) in tumour biopsies predict relapse and survival. We have examined the prognostic significance of TGFα, TGF-$\beta_1$ and EGFR mRNA expression in a series of patients with primary breast cancer with a median follow up period of 60 months. In 167 patients the expression of TGF-$\beta_1$ was inversely correlated with node status ($P = 0.065$) but not ER status, tumour size or menopausal status. Patients with high levels of TGF-$\beta_1$ had a longer disease free interval with a significantly longer probability of survival at 80 months although the overall relapse free survival was not increased. EGFR mRNA expression was measured in 106 patients and was inversely correlated with ER status ($P = 0.018$). EGFR levels did not predict for early relapse or survival. TGFα mRNA levels were measured in 104 patients, no correlation was seen tumour size, node status, Er status, or clinical outcome.

The growth of breast cancer cells is regulated by numerous peptide growth factors including transforming growth factor alpha (TGF-alpha) (Salomon *et al.*, 1984) and transforming growth factor beta-1 (TGF-$\beta_1$) (Knabbe *et al.*, 1987). As breast cancer cells express both the receptor and the ligand for numerous growth factors it has been suggested that an autocrine loop might be an important mechanism of growth regulation for breast cancer *in vivo* (Sporn & Todaro, 1980). TGF-alpha is a 50 amino acid peptide which binds to the Epidermal growth factor receptor (EGFR) and is mitogenic for breast cancer cell lines *in vitro*. TGF-alpha has a more profound and prolonged action on EGFR than Epidermal growth factor itself. It has been reported that TGF alpha synthesis is regulated both *in vitro* (Bates *et al.*, 1988) and *in vivo* (Lui *et al.*, 1987) by oestrogens suggesting a possible role in mediating the effect of endocrine therapy. The importance of a possible autocrine loop in breast cancer involving TGF-alpha is suggested by several studies that have shown a correlation between high levels of EGFR in tumour biopsies and early relapse and survival (Toi *et al.*, 1990; Lewis *et al.*, 1990; Grimaux *et al.*, 1989; Rios *et al.*, 1988; Sainsbury *et al.*, 1987).

The TGF-betas are a family of multifunctional growth factors. The best characterised of these peptides is TGF-$\beta_1$ which is a 25 kDa homodimer which is synthesised by a wide variety of both normal and malignant cells (Derynck *et al.*, 1985). TGF-$\beta_1$ has multiple actions including both stimulatory and inhibitory effects on cell growth although its predominant effect on epithelial cells is inhibitory. It has effects on cellular differentiation, is a chemotactic agent and has numerous effects on extracellular matrix control (Sporn & Roberts, 1989). It has been reported that members of the TGF beta family are regulated *in vitro* by oestrogen (Arrick *et al.*, 1990) and that the anti-oestrogen Tamoxifen increases secretion of locally active TGF beta from breast cancer cells (Knabbe *et al.*, 1987) and stromal fibroblasts (Colletta *et al.*, 1990).

In our preliminary studies of mRNA transcripts of TGF alpha, EGFR and TGF-$\beta_1$ in 69 breast carcinomas with a short follow up of 42.5 months, we found TGF alpha, EGFR, TGF-$\beta_1$ transcripts in 42%, 55% and 100% of tumours respectively and an inverse correlation of EGFR with ER status but, as expected with such a small number of patients and short follow up, no correlation of EGFR, TGF-$\beta_1$ or TGF alpha with prognosis (Barrett-Lee *et al.*, 1990).

We have now extended our observations of clinical correlates with transcript levels of TGF alpha, TGF-$\beta_1$ and EGFR to a larger series of patients with a median follow up time of 60 months.

### Materials and methods

### *Patients and samples*

Breast samples were obtained from the tissue bank kept at the Department of Medical Oncology, St Georges Hospital. In recent years samples have been sent here routinely from several centres for oestrogen receptor analysis after being immediately frozen in liquid nitrogen after resection.

Samples were selected at random from the tissue bank providing the samples had been deposited 5 years previously. They were resected between 1978 and 1984 from patients aged between 36 and 90 years (mean age 62). The clinical details were then obtained from the patients notes. The histological types were infiltrating ductal carcinoma in 149 cases. In addition 11 were lobular carcinomas, two colloid and one medullary carcinomas. Details of T stage and node status were retrospectively extracted from the notes. Any patients found to have another malignancy or a previous breast carcinoma were excluded. Patients who developed a second breast carcinoma were censored at the time of presentation of the second malignancy. The time to relapse was defined as the period from primary surgery to the development of metastatic disease. Patients who relapsed only locally were not considered to have relapsed as local relapse may depend more on the type of primary local therapy administered rather than the biology of the tumour. The median duration of follow up was 60 months (range 6 to 119 months).

### *cDNA probes*

The complementary DNA (cDNA) sequences encoding EGFR (Ullrich *et al.*, 1984), TGF alpha (Derynck *et al.*, 1984), TGF-$\beta_1$ (Derynck *et al.*, 1985) and beta-Actin (Ponte *et al.*, 1984) were excised from plasmids and labelled with alpha-$^{32}$P-dCTP (Amersham, UK) by the random primer method (Feinberg & Vogelstein, 1983).

Total cellular RNA was extracted from 0.5-1 gm of frozen tissue as previously described (Chirgwin *et al.*, 1979). In 10 cases polyadenylated (Poly(A)$^+$) mRNA was obtained by one passage through oligo (dT) cellulose (Aviv & Leder, 1972).

Correspondence: R.C. Coombes, Department of Medical Oncology, Charing Cross Hospital, Fulham Palace Road, London W6 8RF, UK.

To assess the integrity of the RNA extracted all samples were fractionated on Loening phosphategels (Loening & Ingle, 1967) prior to dot-blot analysis. Samples showing evidence of degradation by loss of clear ribosomal bands were discarded. This also served as a check of the concentration of RNA loaded as equal amounts of RNA loaded would fluoresce equally under UV illumination.

For dot blot analysis serial dilutions of denatured RNA were applied to Hybond membranes (Amersham, UK) using a Bio-Dot apparatus (Bio-Rad, UK) as previously described (Barrett-Lee *et al.*, 1987). Serial dilutions of the plasmid being studied and of non-homologous RNA were applied to the membrane to quantify the signal and assess the extent of non-specific hybridisation. For each cDNA probe several tumour samples were analysed by Northern blotting to assess transcript size. For northern analysis (Thomas, 1980) 2.5 μg poly $(A)^+$ or 20 μg total mRNA per sample were resolved in a formaldehyde/agarose gel and blotted onto Hybond-N membrane. Denatured RNA markers were also run to enable sizing of hybridising bands.

Filters were prehybridised at 42°C for 4 h in 50% deionised formamide, 0.1% sodium dodecyl sulphate (SDS), 5 × Denhart's solution (1 × Denhart's = 0.02% each of polyvinylpyrrolidine, bovine serum albumin and Ficoll). Five mM EDTA, 0.75 M NaCl and 50 mM $NaH_2PO_4$ pH 8.3 and denatured salmon sperm DNA (250 $\mu g\ ml^{-1}$).

Filters were hybridised overnight under the same conditions as for prehybridisation with the addition of $1-5 \times 10^6$ c.p.m. $ml^{-1}$ of denatured cDNA probe.

After hybridisation filters were washed with three changes of 2 × SSC (20 × SSC = 3 M NaCl, 0.3 M trisodium citrate, pH7), 0.1% SDS at room temperature and two changes of 0.1 × SSC, 0.1% SDS at 65°C. Autoradiography was carried out using Hyperfilm MP (Amersham, UK) with intensifying screens at −70°C for 4–14 days. To assess the degree of loading of mRNA onto the membranes the membranes were stripped by immersing in 0.1% SDS at 100°C for two hours. Representative filters were then hybridised to the cDNA probe to beta-Actin as described above to ensure that equal loading of RNA had occurred.

Quantification of mRNA was carried out by comparison with serial dilutions of the appropriate plasmid with a scale of + + + + + to + as previously described. (Barrett-Lee *et al.*, 1987). For TGF-$\beta_1$ this was by densitometry while for EGFR and TGF-α the low levels of RNA expression was assessed visually.

### *Oestrogen receptor determination*

Measurement of ER was by a modification of the dextran coated charcoal assay (McGuire & De La Garza, 1973) or alternatively for small samples ER was estimated by an immunocytochemical assay as previously described (McClelland *et al.*, 1986). Using the biochemical assay, carcinomas with > 10 fmol $mg^{-1}$ were considered ER positive while with the immunocytochemical assay, tumours with > 25% staining were considered ER positive.

**Table I** Tumour characteristics related to growth factor expression

| *Tumour characteristics* | | *TGF-α* + *ve* | *TGF-α* − *ve* | *TGF-β₁* *Low* | *TGF-β₁* *High* | *EGFR* + *ve* | *EGFR* − *ve* |
|---|---|---|---|---|---|---|---|
| T stage | T0 | 0 | 1 | 1 | 0 | 1 | 0 |
| | T1 | 7 | 4 | 9 | 23 | 4 | 5 |
| | T2 | 25 | 29 | 29 | 59 | 35 | 32 |
| | T3 | 10 | 9 | 9 | 14 | 6 | 9 |
| | T4 | 4 | 5 | 4 | 6 | 3 | 4 |
| | Tx | 5 | 5 | 4 | 10 | 3 | 5 |
| *Node status* | No | 22 | 24 | 20 | 58 | 20 | 27 |
| | N1 | 17 | 23 | 25 | 37 | 16 | 23 |
| | | | | * | | | |
| *ER status* | − ve | 14 | 18 | 18 | 26 | 10 | 25 |
| | + ve | 28 | 21 | 24 | 40 | 28 | 22 |
| | | | | | | ** | |

*P = 0.064; **P = 0.002.

**Figure 1** **a**, Relapse free survival related to TGF-$\beta_1$ expression. **b**, Survival related to TGF-$\beta_1$ expression.

### *Statistical analysis*

Comparison between subgroups was made with the Chi squared test applying Yates correction were appropriate. Survival analysis was performed using the Log rank test on Life tables. Stratified analysis was made using the Mantel-Cox test on stratified data.

### Results

The clinicopathological details of the patients correlated with expression of TGF-$\beta_1$, TGF-α and EGFR is shown in Table I.

### *TGF-$\beta_1$*

TGF-$\beta_1$ mRNA was measured in 167 carcinomas by dot blot analysis. All tumours contained detectable levels of the transcript. TGF-$\beta_1$ was highly expressed in most tumours. One hundred and twelve tumours (67%) expressed high levels (+ + + + or + + + + + +) while 55 expressed medium or low levels. Northern analysis detected a 2.5 kb transcript. The level of expression of TGF-$\beta_1$ was higher than the expression of TGF-α or EGFR. As there is evidence that oestrogen might regulate the activity and the expression of TGF beta the expression of TGF-$\beta_1$ was examined in relation to ER status. ER status was known in 108 tumours and 64 (59%) were positive. No correlation of TGF-$\beta_1$ with ER status was found ($Chi^2 = .03$ $P = .86$). In 140 patients pathological node status was known. 62 (44%) were node positive. There was an inverse correlation of TGF-$\beta_1$ and node status with high levels of TGF-$\beta_1$ associated with node negativity ($Chi^2 = 3.41$ $P = 0.064$). In 149 patients where menstrual status was recorded there was no correlation of TGF-$\beta_1$ levels with menstrual status ($Chi^2 = 0.01$, $P = 0.92$). There was no correlation of TGF-$\beta_1$ with T stage. We also examined whether there was any correlation of TGF-$\beta_1$ expression with TGF-$\alpha$ or EGFR but no correlation was seen (Data not shown).

The influence of TGF-$\beta_1$ mRNA levels on relapse free survival and survival was examined by life table analysis in 167 patients (Figure 1 a and b). The median follow up of survivors was 60 months. Patients with high levels of TGF-$\beta_1$ had a longer relapse free survival than patients with low levels of TGF-$\beta_1$. The statistical difference in actuarial relapse free survival over the whole period was not significant $P = 0.12$ although the probability of relapse free survival at 80 months was prolonged in the patients with high TGF-$\beta_1$ levels ($P = 0.055$). This suggestion of prolongation of relapse free survival for patients with high TGF-$\beta_1$ levels was accounted for by the relationship with node status as when a correction for node status was applied there was no correlation of TGF-$\beta_1$ levels with relapse free survival ($P = 0.54$, Mantel-Cox test).

### *TGF Alpha*

TGF-$\alpha$ mRNA was measured in 104 tumours by dot blot analysis. Transcripts of 4.8 and 2.2 kb were detected on northern analysis. Levels of expression were low and were scored as present or absent. Hybridisation signals of an intensity less than + of the positive plasmid control were scored as negative. Fifty-three (51%) tumours expressed TGF-$\alpha$ mRNA. In 86 patients where pathological node status was known there was no correlation seen with TGF-$\alpha$ expression ($Chi^2 = .43$ $P = 0.51$). Similarly there was no correlation with ER status in 81 patients where this was known.

There was no evidence that TGF-$\alpha$ mRNA levels had any influence on either relapse free or overall survival (Figure 2a and b).

### *EGFR*

One hundred and six tumours were examined for EGFR mRNA expression by dot-blot analysis. 55 (51%) had detectable levels which were scored as + or + +. Transcripts of 10.0, 6.4 and 4.8 kb were detected by Northern analysis. In 87 tumours the ER status was known and there was a significant inverse correlation of EGFR positivity with ER status. ($Chi^2 = 6.69$ $P = 0.018$). No correlation was seen with pathological nodal status in 86 patients where this was known. From life table analysis there was no evidence from this study in 107 patients with clinical follow up that EGFR mRNA levels predicted either early relapse or death (Figure 3a and b). We found no evidence that co-expression of TGF-$\alpha$ and EGFR was associated with a poor prognosis (data not shown).

### Discussion

This study has addressed the question as to whether the expression (at the mRNA level) of TGF-$\beta_1$, TGF-$\alpha$ or EGFR can have clinical significance. Both epidermal growth factor and TGF-$\alpha$ cause proliferation of breast carcinoma cell *in vitro* and several reports have indicated that levels of EGFR are related to prognosis in breast cancer (Toi *et al.*, 1990; Lewis *et al.*, 1990; Grimaux *et al.*, 1989; Rios *et al.*, 1988; Sainsbury *et al.*, 1987). Other studies have not confirmed this (Foekens *et al.*, 1989; Hawkins *et al.*, 1991) and the exact importance of EGFR levels in breast cancer as a prognostic determinant remains controversial. Our study found an inverse correlation of EGFR with ER status which confirms the reports from other studies (Toi *et al.*, 1990; Lewis *et al.*, 1990; Bolufer *et al.*, 1990; Sainsbury *et al.*, 1988; Battaglia *et*

**Figure 2** **a**, Relapse free survival related to TGF-$\alpha$ expression. **b**, Survival related to TGF-$\alpha$ expression.

**Figure 3** **a**, Relapse free survival related to EGFR expression. **b**, Survival related to EGFR expression.

*al.*, 1988; Rios *et al.*, 1988; Foekens *et al.*, 1989). The correlation with other tumour parameters noted by other workers was not found in this study. A correlation with nodal status (Bolufer *et al.*, 1990), lymphatic invasion (Toi *et al.*, 1990), and tumour size (Sainsbury *et al.*, 1988) has been noted although results have not been consistent. The reason for the discrepancies in the literature might be due to the relatively small size of some of the samples or to differences in methodology of measuring EGFR. Most previous reports have used a ligand binding assay of EGFR with varying levels of cut-off defining EGFR positivity. It can be argued that levels of mRNA might not correlate with EGFR protein levels and this is the reason why no correlation with other parameters has been noted. Although this is possible we have previously shown a high degree of correlation between EGFR mRNA as determined by dot-blot hybridisation and EGFR status as determined by immunocytochemistry (Barrett Lee *et al.*, 1990). The same argument applies to the relationship between mRNA expression and protein expression of TGF-$\beta_1$ and TGF-$\alpha$ but these are not clearly characterised.

As the major ligand for EGFR is TGF-$\alpha$ in breast cancer it is of interest to speculate that tumour levels of TGF-$\alpha$ might effect the natural history of the disease. Recently it has been reported that high levels of TGF-$\alpha$ as measured immunocytochemically correlates with a poor prognosis in adenocarcinoma of the lung (Tateishi *et al.*, 1991). Previous investigators have examined TGF-$\alpha$ expression in small series of patients with breast cancer and have found no evidence of a correlation with ER status, node status or prognosis (Ciardiello *et al.*, 1989; Bates *et al.*, 1988). This is confirmed in our series which is the largest series to date examining this question.

Low levels of TGF-$\beta_1$ mRNA were found to be associated with node positivity and a shorter relapse free survival. TGF-$\beta_1$ is a member of a family of peptides which are highly conserved in nature and are multifunctional. TGF-$\beta_1$ is generally inhibitory to epithelial cells *in vitro* and has several effects on the regulation of extracellular matrix components (Sporn & Roberts, 1989). TGF-$\beta_1$ increases the transcription of the genes for collagen and fibronectin while it decreases the secretion of members of the metalloproteinase family. TGF-$\beta_1$ also increases the production of protease inhibitors including plasminogen activator inhibitor and the tissue inhibitors of metalloproteinase TIMP1 and TIMP2. There is accumulating evidence that the metastatic behaviour of a tumour is strongly determined by its ability to breakdown basement membrane possibly by the increased production of collagenase (Liotta *et al.*, 1991). It is likely that natural tissue proteinase inhibitors would potentially inhibit this invasive process. High levels of TGF-$\beta_1$ may protect against invasion by locally regulating basement membrane components and protease action. TGF-$\beta_1$ is hormonally regulated *in vitro* and several investigators have demonstrated that the anti-oestrogen Tamoxifen increases the secretion of TGF-$\beta_1$ (Knabbe *et al.*, 1987; Colletta *et al.*, 1990) suggesting that this might be an important mediator in the mechanism of endocrine therapy, possibly through the above mechanism.

The potential interplay between TGF-$\beta_1$ and other members of the TGF-$\beta$ family in human breast cancer together with the many known actions of this family of growth factors argues against a simplistic explanation of the role of TGF-$\beta_1$ in this disease. However these data suggest that further studies into the possible role of TGF-$\beta_1$ as a prognostic indicator or as a potential target for novel therapy in breast cancer are warranted.

### References

ARRICK, A.B., KARC, M., DERYNCK, R. (1990). Differential regulation of expression of TGF beta species in human breast cancer cell lines by estradiol. *Cancer Res.*, **50**, 299-303.

AVIV, H. & LEDER, P. (1972). Purification of biologically active globin messenger RNA by chromatography on oligothymidylic acid-cellulose. *Proc. Natl Acad. Sci. USA.*, **69**, 1408-1412.

BARRETT-LEE, P., TRAVERS, M., LUQMANI, Y. & COOMBES, R.C. (1990). Transcripts for transforming growth factors in human breast cancer: clinical correlates. *Br. J. Cancer*, **61**, 612-617.

BARRETT-LEE, P.J., TRAVERS, M.T., MCCLELLAND, R.A., LUQMANI, Y. & COOMBES, R.C. (1987). Characterisation of estrogen receptor mRNA in human breast cancer. *Cancer Res.*, **47**, 6653-6659.

BATES, S.E., DAVIDSON, N.E., VALVERIUS, E.M. & 6 others (1988). Expression of transforming growth factor alpha and its messenger ribonucleic acid in human breast cancer: Its regulation by estrogen and its possible functional significance. *Mol. Endocrinol.*, **2**, 543-555.

BATTAGLIA, F., SCAMBIA, G., ROSSI, S., PANICI, P.B., BELLANTANE, R., POLIZZI, G., QUERZOLI, P., NEGRINI, R., IACOBELLI, S., CRUCITTI, F. & MANCUSO, S. (1988). Epidermal growth factor receptor in human breast cancer: correlation with steroid hormone receptors and axillary lymph node involvement. *Eur. J. Cancer Clin. Oncol.*, **24**, 1685-1690.

BOLUFER, P., MIRALLES, F., RODRIGUEZ, A., VASQUEZ, C., LLUCH, A., GARVIA-CONDE, J. & OLMOS, T. (1990). Epidermal growth factor receptor in human breast cancer: correlation with cytosolic and nuclear ER receptors and with biological and histological tumour characteristics. *Eur. J. Cancer*, **26**, 283-290.

CHIRGWIN, S.M., PRZYBYLA, A.E., MACDONALD, R.J. & RUTTER, W.J. (1979). Isolation of biologically active ribonucleic acid from sources enriched in ribonuclease. *Biochemistry*, **18**, 5294-5299.

CIARDIELLO, F., KIM, N., LISCIA, D.S., BIANO, C., LIDEREAU, R., MERLO, G., CALLAHAN, R., GREINER, J., SZPAK, C., KIDERWELL, W., SCHLOM, J. & SALOMON, D.S. (1989). mRNA expression of transforming growth factor alpha in human breast carcinomas and its activity in effusions of breast cancer patients. *J. Natl Cancer Inst.*, **81**, 1165-1171.

COLLETTA, A.A., WAKEFIELD, L.M., HOWELL, F.V., VAN ROOZENDAAL, K.E.P., DANIELPOUR, D., EBBS, S.R., SPORN, M.B. & GOEDDEL, D.V. (1990). Anti-oestrogens induce the secretion of active transforming growth factor beta from human foetal fibroblasts. *Br. J. Cancer*, **62**, 405-409.

DERYNCK, R., JARRETT, J.A., CHEN, E.Y., EATON, D.H., BELL, J.R., ASSOIAN, R.K., ROBERTS, A.B., SPORN, M.B. & BAUM, M. (1985). Human transforming growth factor-beta, complementary DNA sequence and expression in normal and transformed cells. *Nature*, **316**, 701-705.

DERYNCK, R., ROBERTS, A.B., WINKLER, M.E., CHEN, E.Y. & GOEDDEL, D.V. (1984). Human transforming growth factor alpha: precursor expression and expression in *E. Coli. Cell*, **38**, 287-297.

FEINBERG, A.P. & VOGELSTEIN, B.A. (1983). A technique for radiolabelling DNA restriction endonuclease fragments to high specific activity. *Anal. Biochem.*, **132**, 6-13.

FOEKENS, J.A., PORTENGEN, H., VAN PUTTEN, W.L., TRAPMAN, A.M.A.C., REUBI, J.-C., ALEXIEVA-FIGNSCH, J. & KLIJN, J.G.M. (1989). Prognostic value of receptors for insulin-like growth factor 1, somatostatin, and epidermal growth factor in human breast cancer. *Cancer Res.*, **49**, 7002-7009.

GRIMAUX, M.M., ROMAIN, S., REMVIKOS, Y., MARTIN, P.M. & MAGDELENAT, H. (1989). Prognostic value of epidermal growth factor receptor in node-positive breast cancer. *Breast. Cancer Res. Treat*, **14**, 77-90.

HAWKINS, R.A., KILLEN, E., WHITTLE, I.R., JACK, W.J.L., CHETTY, U. & PRESCOTT, R.J. (1991). Epidermal growth factor receptors in intracranial and breast tumours: their clinical significance. *Br. J. Cancer*, **63**, 553-560.

KNABBE, C., LIPPMAN, M.E., WAKEFIELD, L.M. & 4 others (1987). Evidence that transforming growth factor beta is a hormonally regulated negative growth factor in human breast cancer cells. *Cell*, **48**, 417-428.

LEWIS, S., LOCKER, A., TODD, J.H., BELL, J.A., NICHOLSON, R., ELSTON, C.W., BLAMEY, R.W. & ELLIS, I.O. (1990). Expression of epidermal growth factor receptor in breast carcinoma. *J. Clin. Pathol.*, **43**, 385-389.

LIOTTA, L.A., STEEG, P.S. & STETLER-STEVENSON, W.G. (1991). Cancer metastatic and angiogenesis: An imbalance of positive and negative regulation. *Cell*, **64**, 327-336.

LUI, S.C., SANFILIPPO, B., PERROTEAU, I., DERYNCK, R., SALOMON, D.S. & KIDWELL, W.R. (1987). Expression of transforming growth factor alpha in differentiated rat mammary tumors: estrogen induction of TGF alpha production. *Mol. Endocrinol.*, **1**, 683-692.

MCCLELLAND, R.A., BERGER, U., MILLER, L.S., POWELS, T.J. & COOMBES, R.C. (1986). Immunocytochemical assay for estrogen receptor in patients with breast cancer: relationship to a biochemical assay and to outcome of therapy. *J. Clin. Oncol.*, **4**, 1171-1176.

MCGUIRE, W.L. & DE LA GARZA, M. (1973). Improved sensitivity in the measurement of estrogen receptor in human breast cancer. *J. Clin. Endocrinol. Metab.*, **37**, 986-989.

PONTE, P., NG, S.-Y., ENGEL, J., GUNNING, P. & KEDES, L. (1984). Evolutionary conservation in the untranslated regions of actin mRNAs: DNA sequence of a human beta-actin cDNA. *Nucleic Acids Res.*, **12**, 1687-1696.

RIOS, M.A., MACIAS, A., PEREZ, R., LAGE, A. & SKOOG, L. (1988). Receptors for epidermal growth factor and estrogen as predictors of relapse in patients with mammary carcinoma. *Anticancer Res.*, **8**, 173-176.

SAINSBURY, J.R., FARNDON, J.R., NEEDHAM, G.K., MALCOLM, A.J. & HARRIS, A.L. (1987). Epidermal-growth-factor receptor status as predictor of early recurrence of and death from breast cancer. *Lancet*, **1**, 1398-1405.

SAINSBURY, J.R., NICHOLSON, S., ANGUS, B., FARNDON, J.R., MALCOM, A.J. & HARRIS, A.L. (1988). Epidermal growth factor receptor status of histological sub-types of breast cancer. *Br. J. Cancer*, **58**, 458-460.

SALOMON, D.S., ZWIEBEL, J.A., BANO, M., LASONSZY, I., FEHNEL, P., KIDWELL, W.R. (1984). Presence of transforming growth factors in human breast cancer. *Cancer Res.*, **44**, 4069-4077.

SPORN, M.B. & TODARO, G.J. (1980). Autocrine secretion and malignant transformation of cells. *N. Engl. J. Med.*, **303**, 878-880.

SPORN, M.B. & ROBERTS, A.B. (1989). Transforming growth factor-beta. Multiple actions and potential clinical applications. *JAMA*, **262**, 938-941.

TATEISHI, M., ISHIDA, T., MITSUDOMI, T. & SUGIMACHI, K. (1991). Prognostic implication of transforming growth factor alpha in adenocarcinoma of the lung -an immunocytochemical study. *Br. J. Cancer*, **63**, 130-133.

THOMAS, P.S. (1980). Hybridisation of denatured RNA and small DNA fragments transferred to nitrocellulose. *Proc. Natl Acad. Sci. USA*, **77**, 5201-5205.

TOI, M., NAKAMURA, T., MUKAIDA, H., WADA, T., OSAKI, A., YAMADA, H., TOGE, T., NIIMOTO, M., HATTORI, T. (1990). Relationship between epidermal growth factor receptor status and various prognostic factors in human breast cancer. *Cancer*, **65**, 1980-1985.

ULLRICH, A., COUSSENS, L., HAYFLICK, J.S., DULL, T.J., GRAY, A., TAM, A.W., LEE, J., YARDEN, Y., LIBERMANN, T.A., SCHLESINGER, J., DOWNWARD, J., MAYERS, E.L.V., WHITTLE, N., WATERFIELD, M.D. & SEEBURG, P. (1984). Human epidermal growth factor receptor cDNA sequence and aberrant expression of the amplified gene in A431 epidermoid carcinoma cells. *Nature*, **309**, 418-425.